# Persistent Response and Prolonged Survival Following Pembrolizumab Discontinuation Due to Long-Lasting Autoimmune Colitis in Advanced NSCLC: A Case Report

**DOI:** 10.3389/fonc.2021.670415

**Published:** 2021-06-17

**Authors:** Angela Damato, Loredana De Marco, Silvia Serra, Mario Larocca, Alicia Garcia Arias, Ermanno Rondini, Carmine Pinto

**Affiliations:** ^1^ Medical Oncology Unit, Azienda USL-IRCCS di Reggio Emilia, Reggio Emilia, Italy; ^2^ Department of Medical Biotechnologies, University of Siena, Siena, Italy; ^3^ Pathology Unit, Azienda USL-IRCCS di Reggio Emilia, Reggio Emilia, Italy

**Keywords:** immune checkpoint inhibitors, pembrolizimab, non-small-cell lung cancer, colitis, survival

## Abstract

Pembrolizumab is a programmed death receptor-1 (PD-1) inhibitor that has been approved for treatment of a wide variety of malignancies including non-small-cell lung cancer (NSCLC). Immune-mediated colitis is a known adverse effect of pembrolizumab which can lead to the treatment interruption, although not compromising the control of the oncological disease. Herein, we report the case of a 59-year-old woman on pembrolizumab for advanced NSCLC which developed a severe and persistent colitis treated with infliximab for several months following anti-PD-1 antibody discontinuation. This strategy resulted in an improvement but not complete recovery of the gastrointestinal toxicity despite revealed sustained response and control of the oncological disease with prolonged survival over 24 months.

## Introduction

In NSCLC with programmed death-ligand 1 (PD-L1) expression on ≥50% of tumor cells, first-line treatment with the PD-1 inhibitor pembrolizumab improves survival compared with platinum-doublet chemotherapy (1–3). However, the most gastrointestinal immune-related adverse event (irAE) related to pembrolizumab is colitis and in NSCLC recurred in 1.3% of cases (2, 3). The first choice of treatment for moderate or severe colitis is systemic corticosteroids with symptom improvement, but in some cases it is necessary to consider up-front monoclonal antibody and a discontinuation of immune-checkpoint inhibitor. Despite this, clinical cases of patients with irAEs and sustained response of disease over time after immunotherapy discontinuation are described.

In this clinical case we describe a woman affected by advanced NSCLC treated in first line therapy with pembrolizumab which developed severe colitis requiring symptomatic treatments and simultaneous permanent pembrolizumab discontinuation, keeping a partial response of the oncological disease in the following 24 months, improving the overall survival.

## Case Presentation

This is the case of 59-year-old Caucasian woman from Italy, non-smoker, with no alcohol or drug intake for chronic pathologies. In 2001 she underwent the removal of cystic lymphangioma in the left neck, and in post-surgery she manifested an anaphylactic shock to dexamethasone. In 2008 she was diagnosed with left breast cancer and subjected to bilateral mastectomy, adjuvant chemotherapy, and subsequently, until 2013 she took hormone therapy with tamoxifen.

The oncological history began in December 2017, in which a computed tomography (CT) scan detected a pulmonary lesion in the apical right lobe suspected for neoplasm, bilateral pulmonary micronodules, mediastinal and right para-aortic lymph nodes, and lytic bone lesion in the left sacroiliac synchondrosis. The patient underwent CT-guided needle biopsy of the right lower lobe lesion and histological findings highlighted an adenocarcinoma of pulmonary origin, non-oncogene addicted (EGFR, KRAS and BRAF wild type, ALK and ROS1 not rearranged); PD-L1 tumor proportion score (TPS) expression was positive and equal to 70%. Taking into account the dissemination of the disease and molecular findings (PD-L1 TPS 70%), in January 2018, she started the first line treatment with an anti-PD-1 antibody, pembrolizumab 200 mg at flat dose every three weeks. Therefore, she was exposed to pain-relieving radiotherapy in five fractions (total dose of 20 Gray) on bone lesion.

**Figure 1:**
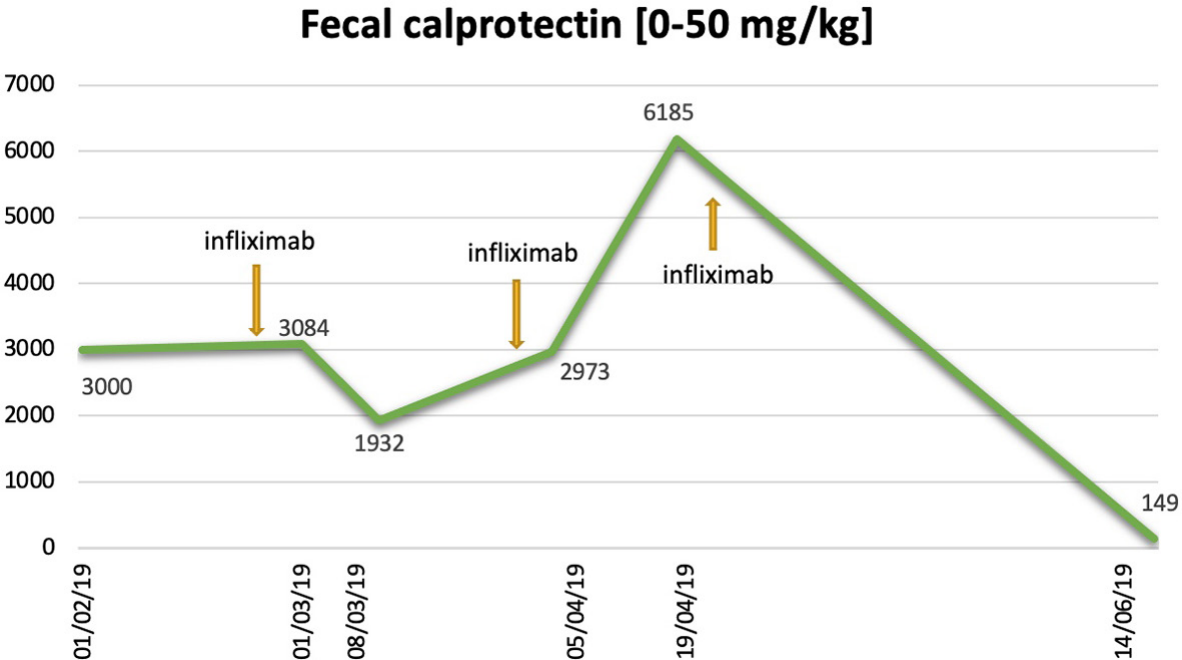
Fecal calprotectin dosage trend and infliximab administrations.

**Figure 2:**
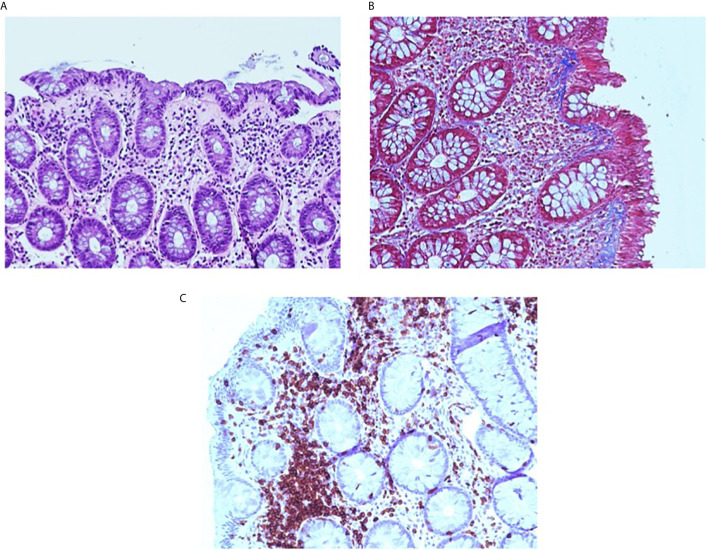
Collagenous colitis pattern. The pink band is seen beneath the surface epithelium and the lamina propria contains increased chronic inflammation **(A)**. Masson trichrome stain highlights the irregularly expanded subepithelial collagen thickening. Note the entrapped inflammatory cells and small vessels **(B)**. CD3 immunostain. The CD3 immunostain confirms that the lymphocytes CD3+ T-cells are predominantly in the lamina propria and are increased **(C)**.

In January 2019, after 1 year of treatment with pembrolizumab, CT scan confirmed the response of disease, with disappearance of the mediastinal lymph nodes and stability of the other disease sites. Despite the excellent response to treatment, after the last administration in January 2019, the patient developed severe grade 4 diarrhea according to Common Terminology Criteria for Adverse Event (CTCAE) version 4.0. For this reason, she was hospitalized and administered intravenous parenteral nutrition and electrolyte supplementation in association with loperamide hydrochloride oral medication. After ruling out a bacterial infection linked to diarrhea such as clostridium difficile, salmonellosis or shigellosis, taken into account the previous heavy steroid allergy resulting in anaphylactic distress, it was not possible to recourse the use of prednisone as treatment of choice for immune-related toxicity. Therefore, in February 2019 an anti-TNF alpha antibody, infliximab 5 mg/kg, was administered with prompt improvement of diarrhea up to grade 2. After 4 weeks, there was a new clinical worsening with abdominal pain, grade 4 diarrhea, weight loss, and cachexia, for which the patient was referred to the emergency department of the hospital and then admitted in our division to start symptomatic cure (intravenous parenteral nutrition, electrolyte supplementation) with further two infliximab administrations, the last in April 2019. The laboratory tests found a remarkable increase of fecal calprotectin in recurrent determinations and variable decrease after infliximab injections (**Figure 1**). To assess the presence of steady, and extensive colonic inflammation, endoscopic assessment with colonoscopy was performed in November 2019 finding marked changes as edematous, thinned, and friability mucosa with diffuse inflammation as an autoimmune colitis. Histopathological features of colon biopsy revealed the mucosa with a pattern of collagenous colitis characterized by the deposition of a subepithelial collagen band and accompanied by inflammatory infiltrate. The lamina propria lymph-plasmacytosis, patchy subepithelial collagen deposition of variable thickness, injury to and detachment of the surface epithelium, and glandular atrophy were seen (**Figure 2**). From June 2019 to December 2019, further six infliximab administrations were given up to decrease the intensity of diarrhea to grade 1. Despite repeated administration of infliximab, no clinical signs of infection were found. At follow-up of 10 months after the last infliximab administration, the medical conditions of patient revealed weight recovery, occasional abdominal pain, and grade 1 diarrhea.

**Figure 3:**
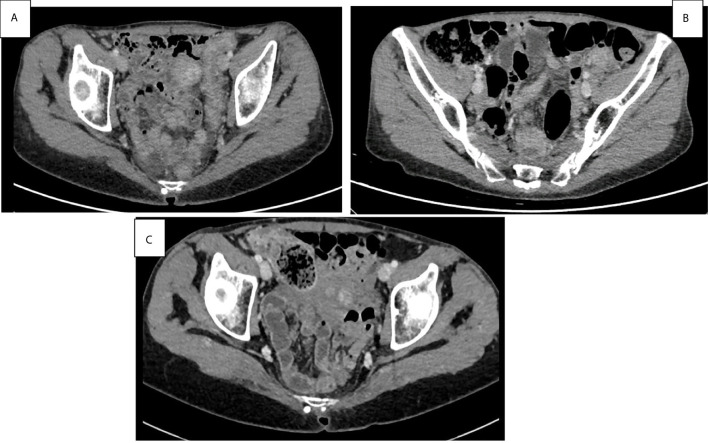
Basal abdominal CT scans at the onset symptoms of pan-colitis with diffuse thickening of the colon walls **(A)**. Improvement of pan-colitis after 8 months of infliximab treatment and 9 months of Pembrolizumab discontinuation **(B)**. Persistent mild colitis after 24 months of Pembrolizumab discontinuation **(C)**.

**Figure 4:**
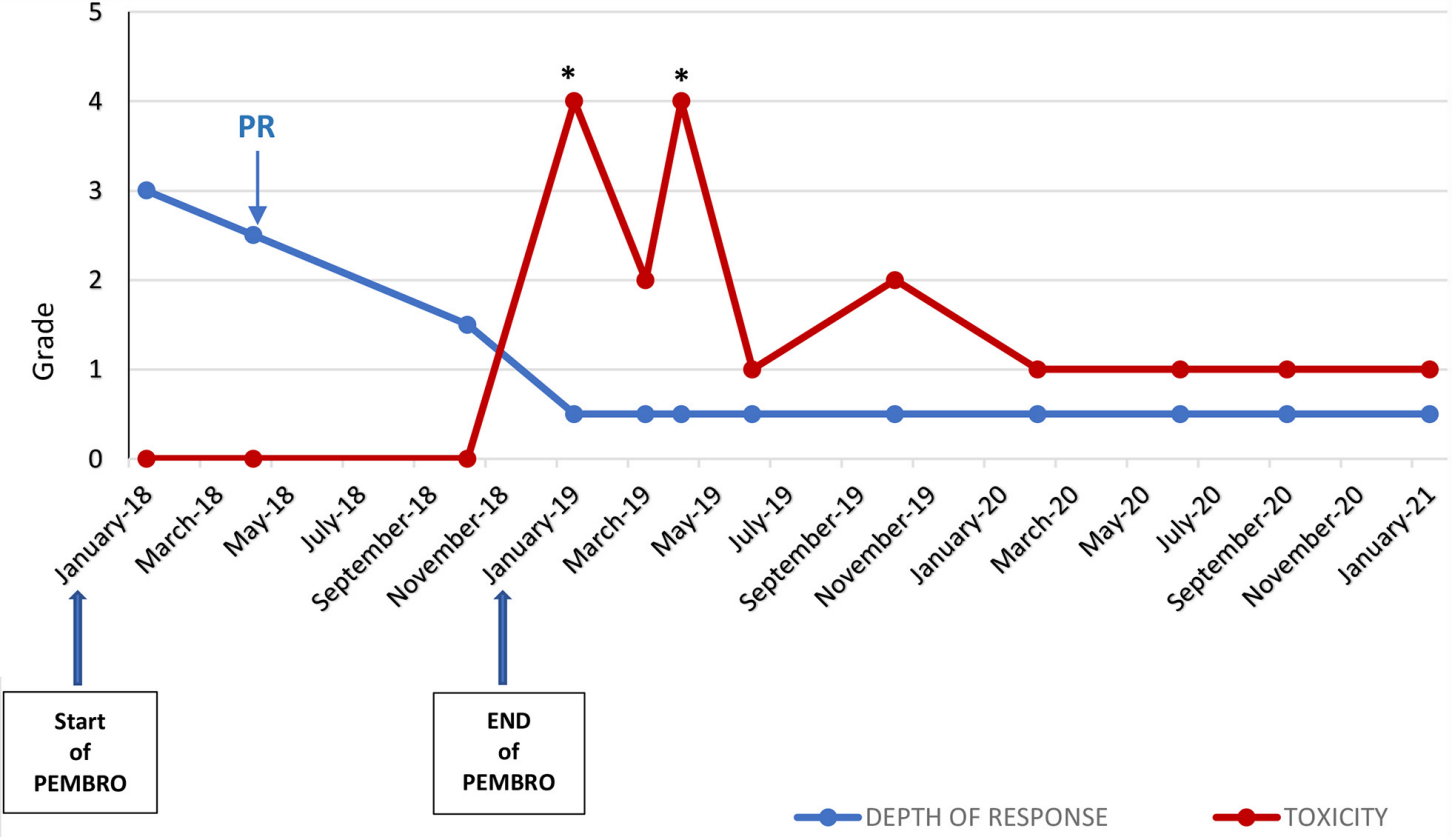
Trend over time of the radiological response to pembrolizumab by RECIST Criteria version 1.0 and grade of gastrointestinal irAE (diarrhea) by CTCAE version 4.0 *Hospitalization; PD, Partial Response (according to RECIST v1.0); PEMBRO, pembrolizumab.

The instrumental follow-up with CT scan achieved in January 2021, 24 months after the latest administration of pembolizumab (carried out on January 2019), showed a persistent and remarkable stability of oncological disease in lung, lymph-nodes, and bone, despite the widespread inflammation of the colon *in toto* described as bowel wall thickening and colonic distension (**Figure 3**). The trend over time of the radiological response to immunotherapy and gastrointestinal toxicity evolution is shown in **Figure 4**.

## Discussion

The immunotherapy era has meaningfully improved cancer management and survival outcomes, mostly in patients with NSCLC (1–3). In first-line setting, pembrolizumab as monotherapy alone or in combination with chemotherapy improved long-term outcomes. In the phase I KEYNOTE-001 study, pembrolizumab improved clinical outcomes in patients with advanced NSCLC PD-L1 TPS ≥50% treated compared with tumors with lower PD-L1 levels (1). As a result, a PD-L1 expression level of ≥50% was selected for the KEYNOTE-024 study, a randomized phase III trial which demonstrated prolonged overall survival (OS) in first-line with pembrolizumab compared with platinum doublet chemotherapy for advanced NSCLC (2). At the median follow-up of 5 years, the median OS (mOS) was 26.3 months *versus* 13.4 months with chemotherapy [CI 95%, HR 0.62 (0.48–0.81)] and overall response rate (ORR) of 32% (3). Furthermore, in the phase III KEYNOTE-042 study, pembrolizumab alone compared to chemotherapy in first line setting, according to the PD-L1 TPS level 1–19, 20–49, and ≥50% revealed a median OS improvement of 16.7, 17.7, and 20.0 months, respectively in each subgroup (4).

Because of the synergy between chemotherapy and immunotherapy, the first-line combined treatment based on pembrolizumab is another option in non-squamous NSCLC as suggested by KEYNOTE-189 phase III study (5). However, an unresolved question is whether to use pembrolizumab monotherapy or pembrolizumab plus chemotherapy in patients with PD-L1 level ≥50%. It is necessary to identify biomarkers to select patients who respond to pembrolizumab in monotherapy and spare patients the added toxicities of chemotherapy.

The immune check point inhibitors (ICIs) are involved in the downregulation of cytotoxic T cells, stimulating cytotoxic T-cell survival, strengthening of tumor surveillance and antitumor action. Despite these activities, ICIs also trigger global T-cell responses that prompt several immune-related adverse events (irAEs), of which the most serious and clinically relevant is colitis (6, 7). One of the suggestive symptoms is diarrhea defined as loose, watery stools a day that occurs in 12.1–13.7% and colitis associated to presence of abdominal pain, rectal bleeding, and mucous in the stools of patients treated with anti-PD-1 antibody (8–10). Colitis is defined by endoscopically mucosal ulcerations or fecal calprotectin dosage. Moreover, stools should be checked for bacterial, parasitic, and viral infections including *Clostridium difficile* (11–13). A widespread and detailed history, physical examination, and early endoscopic assessment are encouraged to diagnosis and make a prognosis of immune-mediated colitis when immunotherapy is considered (14). Mucosal ulcerations are present in 30–40% of cases, whereas in 35–40% of cases edema, exudate, unusual vascularity, and erosions were found (15). These features demand systemic therapy and hospitalizations to control symptoms and electrolyte imbalance. Systemic corticosteroids such as prednisone 1–2 mg/kg are the first-line approach for irAEs and described to be effective in 87.5% of patients (16). Once clinical improvement to grade 1 or less is achieved, steroids should be progressively reduced, and anti-PD-1/L1 inhibitors can usually be resumed when symptoms have resolved or prednisone is tapered to daily doses of 10 mg or less. The risk of recurrent gastrointestinal irAEs is reported as high as 19–36% (17). Cases of persistent inflammation up to 6–18 months from initial diagnosis (18) are described, as in our clinical case. Although we performed the intestinal biopsy about 10 months after the drug discontinuation, it is likely that what was seen corresponds to the outcome of a collagenous colitis linked to the intake of the anti PD-1 antibody. An escalation of biologic agents is recommended for steroid-refractory immune-colitis or those who cannot use the steroids as a therapy. Infliximab, a tumor necrosis factor (TNF)-alpha antagonist, is effective with faster symptom resolution in a median of 3 days (18). This inhibition enhances tumor immunity by facilitating the proliferation and function of T-regs and myeloid-derived suppressor cells, overcoming resistance to anti–PD-1 antibodies (19). Furthermore, after infliximab exposure, the risk of immunogenicity due to sporadic dosing should be considered for patients with recurrent disease, with potential infusion reactions or weakening effectiveness. Apart from this, infliximab therapy influences the gut microbiota dysbiosis by modifying microbiota composition and function, especially in Chron’s disease, highlighting a reduction in pathogenic bacteria such as *Fusobacterium*, *Enterobacter*, and *Escherichia–Shigella*, and an increase in short-chain fatty acid-producing bacteria such as the family Lachnospiraceae (20). Although in the cancer setting there is little evidence, this gut microbioma could be a biomarker for monitoring response to treatment.

Few and discordant data exist regarding the clinical outcomes in advanced NSCLC following immunotherapy interruption due to irAEs. In a retrospective analysis of nivolumab-treated patients with advanced NSCLC who developed colitis had a lower median OS compared to those who did not (4.4 *vs*. 10.6 months, P= .010) (21). Conversely, in a series of retrospective studies including patients with metastatic renal-cell carcinoma who discontinued PD-1 or PD-L1 antibodies after an initial response due to irAEs revealed a prolonged time to progression (22). A recent large real-world analysis of patients with NSCLC with PD-L1 expression ≥50%, treated with single-agent pembrolizumab including frail patients showed a significant association between irAE occurrence and improved PFS, except for gastrointestinal irAEs not associated with an improved ORR and OS. The authors concluded that irAE occurrence may be a surrogate of clinical activity and improved outcomes in this setting (23). Naquash et al. conducted a pooled exploratory analysis of 531 patients with advanced NSCLC treated with nivolumab derived from five retrospective cohorts showing an improved PFS and OS in patients that had irAEs during the treatment (24). At last, a record of 1,959 patients treated with nivolumab in an Italian NSCLC expanded access program, confirmed a significantly higher response rate, disease control rate, mPFS, and mOS in patients developing irAE of any grade (25).

These considerations might differ depending on tumor types, treatment protocols, and may be, by the physicians’ experience in reporting irAEs and its management. Supporting the data of the retrospective studies, our clinical case revealed, 24 months from immunotherapy discontinuation due to the onset of colitis, a sustained response and control of the oncological disease with prolonged survival in line with the retrospective cases reported above.

## Conclusions

Pembrolizumab-induced immune-mediated colitis can occur in patients with NSCLC. Accurate diagnosing of immunotherapy-related colitis is mandatory, by acting with systemic and appropriate care. The first choice of treatment to counteract the symptoms are steroidal anti-inflammatory drugs (SAIDs) however, in some peculiar cases, patients may require biological therapy as anti-TNF-alpha antibody. It is worth noticing that pharmacological therapy may not be sufficient to control the gastrointestinal irAEs and a prolonged and conclusive immunotherapy discontinuation is necessary. Interestingly, the clinical outcome in such patients has been under investigation and in our clinical case, we report a remarkable and prolonged response of the oncological disease, which is maintained over time impacting positively on patient’s survival. Further analysis should investigate the interplay between immune-mediated colitis and survival, and also biological studies of correlation with toxicities.

## Data Availability Statement

The raw data supporting the conclusions of this article will be made available by the authors, without undue reservation.

## Ethics Statement

The studies involving human participants were reviewed and approved by AUSL—IRCCS Reggio Emilia. The patients/participants provided their written informed consent to participate in this study. Written informed consent was obtained from the individual(s) for the publication of any potentially identifiable images or data included in this article.

## Author Contributions

AD, ER, ML, and AA interacted with the patient. AD wrote the manuscript. LM and SS prepared and analyzed pathology. All authors contributed to the article and approved the submitted version.

## Conflict of Interest

The authors declare that the research was conducted in the absence of any commercial or financial relationships that could be construed as a potential conflict of interest.
